# NO_x_-Free Leaching Methods for Efficient Silver and Aluminium Recovery from Crystalline Silicon Solar Cells

**DOI:** 10.3390/ma18112668

**Published:** 2025-06-05

**Authors:** Aistis Rapolas Zubas, Egidijus Griškonis, Gintaras Denafas, Vidas Makarevičius, Rita Kriūkienė, Jolita Kruopienė

**Affiliations:** 1 Institute of Environmental Engineering, Kaunas University of Technology, Gedimino St. 50, LT-44239 Kaunas, Lithuania; jolita.kruopiene@ktu.lt; 2 Department of Environmental Technology, Faculty of Chemical Technology, Kaunas University of Technology, Radvilėnų pl. 19, LT-50254 Kaunas, Lithuania; gintaras.denafas@ktu.lt; 3 Department of Physical and Inorganic Chemistry, Faculty of Chemical Technology, Kaunas University of Technology, Radvilėnų pl. 19, LT-50254 Kaunas, Lithuania; egidijus.griskonis@ktu.lt; 4 Laboratory of Materials Research and Testing, Lithuanian Energy Institute, Breslaujos St. 3, LT-44403 Kaunas, Lithuania; vidas.makarevicius@lei.lt (V.M.); rita.kriukiene@lei.lt (R.K.)

**Keywords:** solar cell recycling, photovoltaic waste management, hydrometallurgical leaching, silver, aluminium, NO_x_-free processes

## Abstract

As photovoltaic (PV) installations expand globally, effective recycling of end-of-life crystalline silicon solar cells has become increasingly important, including the recovery of valuable metals such as silver (Ag) and aluminium (Al). Traditional nitric acid-based chemical leaching methods, although effective, present environmental challenges due to the generation of hazardous nitrogen oxide (NO_x_) emissions. To address these concerns, this study investigated alternative hydrometallurgical leaching strategies. Two selective treatments (NaOH for Al, and NH_3_ + H_2_O_2_ for Ag) and one simultaneous treatment (HNO_3_ + H_2_O_2_) were evaluated for metal recovery efficiency. All methods demonstrated high recovery efficiencies, achieving at least 99% for both metals within 60 min. The investigated methods effectively suppressed NO_x_ emissions without compromising leaching efficiency. These findings confirm that hydrometallurgical leaching techniques incorporating hydrogen peroxide can achieve efficient and environmentally safer recovery of silver and aluminium from solar cells, providing valuable insights into the development of more sustainable recycling practices for photovoltaic waste management.

## 1. Introduction

The transition from fossil fuels to renewable energy systems has led to a rapid global increase in photovoltaic (PV) installations [1]. Although advances in optoelectronic [2] and absorber materials [3] continue to be reported, PV remain particularly based on crystalline silicon (c-Si) technologies [4]. They have dominated the market with an 80–90% share over the last 40 years and thus will lead the domination of PV waste stream as well [5]. In the European Union, c-Si modules waste is predicted to be 80–87% of overall PV wastes during 2020–2050 [6]. It is estimated that globally, photovoltaic wastes will exceed 60–78 million tonnes by 2050 [5,7]. A typical PV panel is dominated by glass but their embedded solar cells contain highly valuable and resource-intensive materials such as silver (Ag), silicon (Si), and aluminium (Al), which are increasingly targeted in recycling efforts [8,9]. Mechanical, thermal, and chemical methods are employed to delaminate PV module in order to remove an encapsulating polymer for the separation of glass, solar cells, and metal ribbons [10]. Then, chemical approaches are utilised for the leaching of silver and aluminium from the surface of silicon solar cells [11]. Silver paste consisting of approximately 90% of silver is screen printed onto the front side of the cell in the form of fine conductive fingers and busbars [12]. Aluminium is typically applied to the entire rear surface of the cell, serving as the back contact field [13]. Silver, though present in small quantities (approximately 0.05% by weight), accounts for a major fraction of a cell’s value due to its high price and critical role in the metallization of c-Si cells [14]. Aluminium found in metal frame and solar cells is also of economic interest, with the added benefit of being easily and energy efficiently recyclable [15]. Silver and aluminium represent the highest economic value among recoverable materials from waste photovoltaic modules, accounting for approximately 47% and 26%, respectively [16].

Unlike whole module recycling, which also addresses recovery of glass, solar cell recycling focuses specifically on high value recovery of metals. Hydrometallurgical approaches have gained attention due to their potential for effective metal recovery from solar cells [17,18,19,20,21,22,23,24,25,26,27,28]. Single step or multistep approaches are employed to leach silver and/or aluminium from solar cells with selective one metal in solution or both. Traditional method frequently relies on nitric acid (HNO_3_) due to its strong oxidising capacity and high selectivity for silver. Leaching typically involves immersing shredded or pretreated solar cells in HNO_3_ (1–4 M) at elevated temperatures (60–90 °C) to dissolve silver into solution [12,17,19,23,26,28]. It is often followed by treatment with hydrochloric acid (HCl) to form silver chloride (AgCl) [18,29,30]. The efficiency of this process is high, with reported silver recovery yields exceeding 95%; however, this method has a major drawback in generating hazardous nitrogen oxides (NO_x_), contributing significantly to environmental pollution [17,18,27,30].

Aluminium recovery from solar cells often involves alkaline leaching, with sodium hydroxide (NaOH) as a common reagent. The amphoteric nature of aluminium allows it to dissolve readily in NaOH solutions, typically in concentrations ranging from 0.5 M to 4 M and temperatures between 40 and 80 °C [17,29,31]. This method is favoured for its selectivity and low environmental impact, and aluminium can be subsequently recovered by pH-controlled precipitation [31]. While this method is efficient for aluminium recovery, it results in hydrogen gas evolution, presenting potential safety concerns and requiring controlled ventilation or gas capture systems [29]. Nitric acid can also be used for the leaching of aluminium from solar cells, although its effectiveness is generally lower compared to alkaline solutions [19,26]. Aluminium dissolution in nitric acid typically occurs at higher acid concentrations and temperatures, contributing to dissolution of both metals during single step leaching processes [17,23,24]. Sequential leaching processes combining NaOH and HNO_3_ have also been proposed, with NaOH used to extract aluminium first, followed by nitric acid treatment to dissolve silver [17,29,31]. While this approach enhances metal selectivity and recovery, the primary drawback of nitric acid remains, i.e., the generation of environmentally hazardous nitrogen oxides, which necessitate careful gas handling and mitigation systems. Several NO_x_-free approaches have previously been explored for the leaching of silver from crystalline silicon solar cells, including methods employing sulfuric acid (H_2_SO_4_) with sonication [25] or organic solution such as C_6_H_10_O_8_ combined with hydrogen peroxide (H_2_O_2_) [32]. While these methods effectively eliminate NO_x_ emissions, potential limitations related to operational costs or process complexity persist.

In this context, our study presents novel hydrometallurgical recycling approaches for solar cells. By utilising alternative reagents, our methods enable the effective leaching of silver and aluminium while avoiding the generation of nitrogen oxides. Ammonia with hydrogen peroxide (NH_3_ + H_2_O_2_) was chosen for selective leaching of silver from the solar cells. It is an environmentally friendly leaching method suitable for specific metals such as silver, but not aluminium. Metallic silver does not dissolve in plain ammonia, but in the presence of an oxidant like H_2_O_2_, it is readily leached by forming a soluble diammine silver (I) complex. Operating under mild, basic conditions, this method offers an effective alternative to acid-based processes without generating nitrogen oxide emissions [33,34].

A universal method for simultaneous silver and aluminium recovery was also applied. Nitric acid is extensively reported in the literature as an effective leaching agent for these metals. However, its use is commonly associated with the generation of NO_x_. The incorporation of hydrogen peroxide into the leaching system not only enhances oxidative dissolution but also mitigates NO_x_ emissions by promoting the reoxidation of intermediate nitrogen species to nitric acid. H_2_O_2_ acts as an oxidant and oxygen source in acidic media and can react with nitric oxide (NO) or nitrogen dioxide (NO_2_) to regenerate nitric acid in situ [35]. In a nitric leach, any NO or NO_2_ emitted from metal dissolution is immediately oxidised by H_2_O_2_ back into HNO_3_. Nitric acid is regenerated, reducing the amount of fresh acid needed. This approach offers a more controlled and environmentally sustainable alternative for metal recovery from various multilayer products, including photovoltaic solar cells.

This study aims to present NO_x_-free hydrometallurgical leaching approaches for recovering silver and aluminium from crystalline silicon solar cells, introducing methods not previously reported in photovoltaic recycling literature. By performing these alternative strategies, the research contributes to the development of more sustainable PV recycling technologies.

## 2. Materials and Methods

The solar cells used in this study were obtained from frameless Solitek Solid Pro 300 W M.60 glass/glass PV module. The pretreatment was carried out to obtain the solar cells for the trials. First, the junction box was manually removed from the back side of the module. Then, the module was cut into smaller segments using an angle grinder to allow insertion into an electric muffle furnace. Thermal treatment was applied at 500 °C for one hour in ambient air. This method is sufficient to achieve effective separation of the module layers such as glass, solar cells, and metal ribbons [36]. Following thermal delamination, encapsulated solar cells were manually separated and collected for hydrometallurgical treatment aimed at silver and aluminium recovery.

Due to the mechanical cutting performed during sample preparation, the solar cells were already fragmented into smaller pieces. However, certain fragments remained relatively large and were further reduced in size by manual crushing using a porcelain mortar and pestle. The material was not pulverised to a fine powder, as fractionation was not required for this stage of the study.

Two distinct hydrometallurgical leaching approaches were applied to the crushed solar cells. All leaching trials were performed in a self-made laboratory reactor (max. volume 3 L) equipped with integrated controlled heating and stirring functions.

### 2.1. Selective Leaching of Aluminium with Sodium Hydroxide (NaOH) and Silver with Ammonia and Hydrogen Peroxide (NH_3_ + H_2_O_2_)

#### 2.1.1. Aluminium Leaching with Sodium Hydroxide (NaOH)

A total of 100 g of crushed solar cells was immersed in 2 L of sodium hydroxide solution (50 g/L concentration), by a solid/liquid ratio of 1:20. The reaction was carried out at a temperature of 40 °C. After 30 min of leaching, a portion of the samples was removed for interim analysis, while the remaining solar cells continued leaching for another 30 min. After, the samples were removed, rinsed with distilled water, and dried in an oven at 105 °C for 2 h.

#### 2.1.2. Silver Leaching with Ammonia and Hydrogen Peroxide (NH_3_ + H_2_O_2_)

Dried samples from the first step were subjected to silver leaching. A 1 L solution was prepared consisting of 125 mL of 25% NH_3_, 750 mL of distilled water, and 125 mL of 35% H_2_O_2_, achieving a solid/liquid ratio of 1:10. The leaching process was conducted at room temperature without external heating. Due to the high reactivity of hydrogen peroxide, it was divided in four equal portions of 31.3 mL each: the first was added at the start, and the remaining three were added at 15 min intervals. As with aluminium leaching, some samples were removed after 30 min and the rest after 60 min, followed by washing and drying at 105 °C for 2 h.

### 2.2. Simultaneous Leaching of Aluminium and Silver with Nitric Acid and Hydrogen Peroxide (HNO_3_ + H_2_O_2_)

In the second approach, both metals were leached simultaneously. A total of 100 g of crushed solar cells was immersed in 1 L of mixed acid solution comprising 200 mL of 65% nitric acid, 600 mL of distilled water, and 200 mL of 35% hydrogen peroxide, maintaining a solid/liquid ratio of 1:10. The leaching was conducted at 50 °C. Hydrogen peroxide was again added gradually in four equal portions of 50 mL each: the first at the start of the trial and the others at 15 min intervals. Sampling, washing, and drying procedures were consistent with the previous trials.

To evaluate leaching efficiency, the surface structure of the solar cells, chemical composition, and extracted products were analysed using a ZEISS EVO MA10 scanning electron microscope (SEM) with a Bruker XFlash 6/10 EDS detector, employing energy-dispersive X-ray spectroscopy (EDS). Analyses were performed on the front and back surfaces of untreated solar cells to establish baseline elemental concentrations. The silver content was assessed by analysing silver fingers on the front side, while aluminium was evaluated from the back surface layer. Samples taken after 30 and 60 min of leaching were analysed under identical conditions. Leaching efficiency (E) was calculated based on the relative reduction in elemental content observed via SEM-EDS and is expressed as a percentage:

(1) $$E=\frac{C_{0}-C_{t}}{C_{0}}\times 100\%$$

where *C*_0_ is the initial atomic percentage of the element (Ag or Al) on the surface prior to leaching and *C_t_* is the atomic percentage of the same element after a specified leaching time.

This approach provides a direct estimate of how much of the target metal has been removed during the metallurgical treatment process of the solar cells.

## 3. Results

### 3.1. SEM-EDS Analyses of Untreated Solar Cells

**Figure 1:**
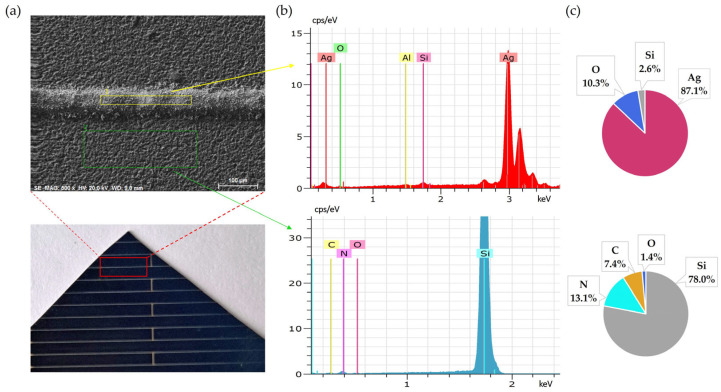
Front side of solar cell before leaching: (**a**) SEM image, (**b**) EDS spectra, (**c**) elemental concentration.

**Figure 2:**
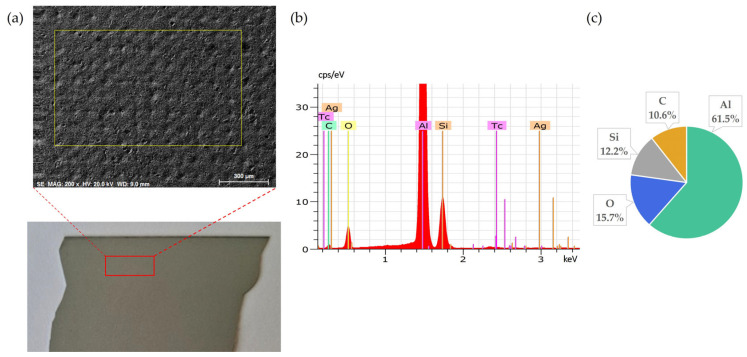
Back side of solar cell before leaching: (**a**) SEM image, (**b**) EDS spectrum, (**c**) elemental concentration.

Initial SEM-EDS analyses of untreated solar cell surfaces are presented in Figure 1 and Figure 2. Figure 1 shows the elemental composition of the front side silver fingers, clearly illustrating the differences between the silver-coated (No. 1/yellow) and non-coated areas (No. 2/green) of the cell surface. Peaks and atomic percentage distributions confirm a dominant presence of silver (Ag) on the metallic fingers, while the non-coated regions primarily consist of silicon (Si).

Figure 2 presents the elemental composition of the back side of solar cells, confirming that Al is the dominant element, as expected due to its use as the back surface contact layer. The clear coverage of aluminium confirms the suitability of this surface for the targeted leaching tests.

### 3.2. Selective Leaching of Aluminium and Silver

#### 3.2.1. Aluminium Leaching

Aluminium leaching with NaOH was employed as a first step for selective metal removal. The leaching effectiveness of aluminium from the back side of the solar cells was assessed by visual inspection and SEM-EDS analysis. Before leaching, the back surface was fully covered with an aluminium layer. After placing the solar cells into a NaOH solution, visible gas evolution of hydrogen (H_2_) occurred as a result of the reaction between aluminium and sodium hydroxide:

Al_2_O_3_ + 2NaOH + 3H_2_O → 2Na[Al(OH)_4_] (2)

2Al + 2NaOH + 6H_2_O → 2Na[Al(OH)_4_] + 3H_2_ (3)

This exothermic reaction caused a slight temperature increase, reaching approximately 44 °C. It is important to highlight that due to hydrogen’s flammability and potential explosiveness, adequate ventilation or gas capture and subsequent decontamination are recommended for safe scale-up or industrial implementation.

After 30 min, primary visual inspection showed partial removal of aluminium from the surface—some areas were clean of aluminium, while others were still covered with the metal layer. Extending the reaction time to 60 min further reduced aluminium coverage. The metallic silicon surface after the trial was visible; then the solar cells were removed from the solution and washed. However, longer exposure was avoided as it initiates a reaction between NaOH and Si under the following reactions:

SiO_2_ + 2NaOH → Na_2_SiO_3_ + H_2_O (4)

Si + 2NaOH + H_2_O → Na_2_SiO_3_ + 2H_2_ (5)

Longer exposure would lead to silicon etching, which is undesirable in the context of this study. Thus, a leaching duration of approximately 60 min was determined as optimal to effectively remove the aluminium back contact while minimising potential etching of silicon wafer.

#### 3.2.2. Silver Leaching

The second step involved selective removal of silver from the front side of the solar cells using ammonia combined with hydrogen peroxide (NH_3_ + H_2_O_2_). This chemical mixture reacts with silver, forming stable silver–ammonia complex ions.

When the solar cells were immersed in the solution, the reaction started quickly and visibly, leading to noticeable bubbling and an increase in solution temperature from the initial room temperature (22 °C) to approximately 43 °C within the first 23 min. This temperature rise was caused by the chemical reaction among metallic silver, ammonia, and hydrogen peroxide, forming soluble silver–ammonia complexes according to the following reaction:

2Ag + 4NH_3_ + H_2_O_2_ → 2[Ag(NH_3_)_2_]OH (6)

**Figure 3:**
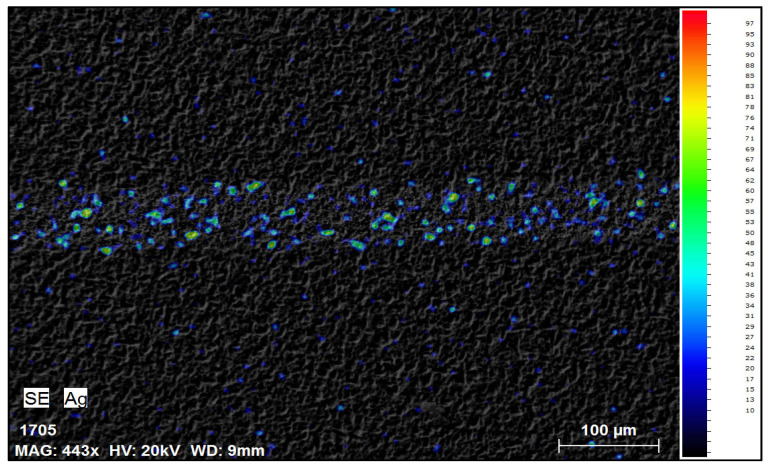
SEM-EDS mapping image of solar cell front side after 30 min in NH_3_ + H_2_O_2._

After the first 30 min of reaction, visual inspection under a microscope showed significant removal of silver from the cell surface. The previously continuous silver fingers appeared mostly dissolved, leaving behind faint metallic traces in some locations. SEM-EDS analyses conducted after drying the samples provided additional confirmation, clearly demonstrating that the majority of silver was removed after 30 min of trial; however, some traces of silver could be observed (Figure 3).

**Figure 4:**
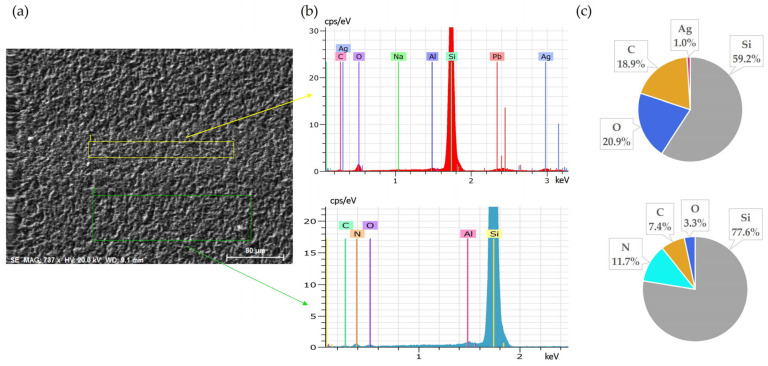
Front side of solar cell after 60 min leaching in NH_3_ + H_2_O_2_: (**a**) SEM image, (**b**) EDS spectrum, (**c**) elemental concentration.

Continuing the leaching for an additional 30 min (60 min total) resulted in nearly complete removal of silver from the surface, as confirmed by SEM-EDS analysis (Figure 4). This further improvement illustrates slightly improved effectiveness of longer exposure.

In addition to the high efficiency of silver dissolution, this ammonia-based method offers the advantage of avoiding harmful NO_x_ emissions, providing a safer alternative to conventional nitric acid-based silver leaching methods. Furthermore, the formation of the silver–ammonia complex not only helps dissolve silver but also prevents silver reprecipitation on the silicon surface, maintaining high purity of recovered materials and simplifying downstream processing.

### 3.3. Simultaneous Leaching of Aluminium and Silver with Nitric Acid and Hydrogen Peroxide

The third method aimed to leach aluminium and silver simultaneously using a solution of nitric acid combined with hydrogen peroxide. According to the literature, nitric acid is commonly used to remove metals from solar cells due to its strong oxidising properties, which help dissolve metallic layers effectively. These properties make nitric acid one of the most preferred leaching agents in hydrometallurgical solar cell recycling. In this study, hydrogen peroxide was included to specifically prevent the formation of NO_x_ (in the case of concentrated acid, mainly NO_2_; and in the case of diluted nitric acid, mainly NO), which are typically generated when nitric acid alone is used.

When solar cells were placed into the HNO_3_ + H_2_O_2_ solution, the reactions initiated actively, indicating rapid oxidative interactions between metals and leaching agents. The main chemical reactions responsible for aluminium and silver leaching are presented below:

Aluminium:

Al + 4HNO_3_ → Al(NO_3_)_3_ + NO + 2H_2_O—first stage (7)

2NO + 3H_2_O_2_ → 2HNO_3_ + 2H_2_O—second stage (8)

2Al + 6HNO_3_ + 3H_2_O_2_ → 2Al(NO_3_)_3_ + 6H_2_O—net reaction (9)

Silver:

3Ag + 4HNO_3_ →3AgNO_3_ + NO + 2H_2_O—first stage (10)

2NO + 3H_2_O_2_ → 2HNO_3_ + 2H_2_O—second stage (11)

2Ag + 2HNO_3_ + H_2_O_2_ → 2AgNO_3_ + 2H_2_O—net reaction (12)

**Figure 5:**
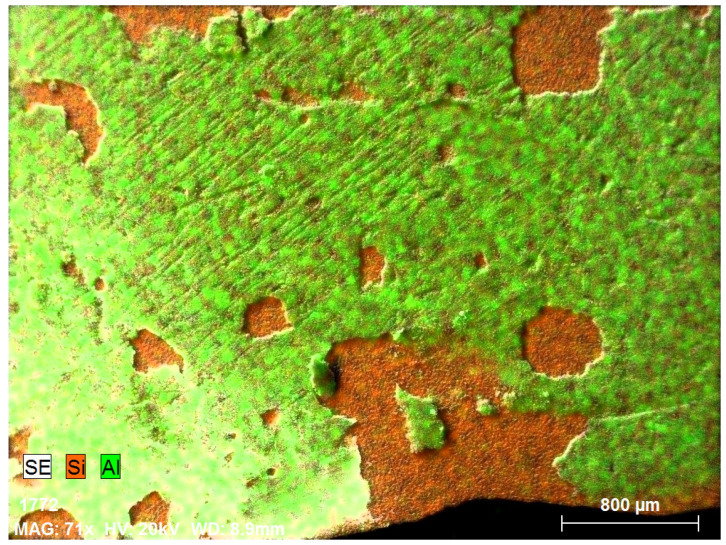
SEM-EDS mapping image of solar cell back side after 30 min in HNO_3_ + H_2_O_2._

The samples removed from the solution after the first 30 min showed partial but uneven removal of aluminium and silver from solar cells. For Al, the initial removal was visibly uneven, with certain areas of the cell surface showing clear signs of dissolution, while others remained largely covered by aluminium (Figure 5). SEM-EDS analysis quantified this partial removal, indicating Al leaching efficiency of approximately 79% after 30 min.

Similarly, silver leaching was incomplete after the same duration. Residual metallic silver was clearly visible under microscopic inspection on the front side metal fingers. They were more prominent compared to the results from selective Ag leaching with NH_3_ + H_2_O_2_, suggesting initially slower leaching.

**Figure 6:**
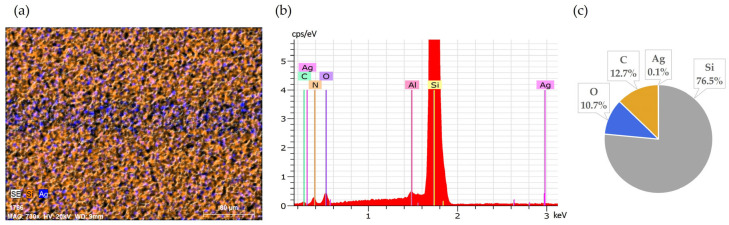
Front side of solar cell after 60 min leaching in HNO_3_ + H_2_O_2_: (**a**) SEM image, (**b**) EDS spectrum, (**c**) elemental concentration.

Extending the leaching duration to 60 min significantly improved the overall leaching results. SEM-EDS analyses confirmed nearly complete removal of both Al and Ag. After 60 min, silver fingers exhibited minimal remaining Ag content (Figure 6), and Al was similarly removed more uniformly from the back surface, demonstrating that a longer reaction time compensated the lower leaching efficiency at the beginning.

Throughout the entire process, the reaction between the solar cells and the leaching solution remained active. Importantly, no visible emission of brownish gases was observed, indicating the successful prevention of NO_x_ formation. This method confirmed the high efficiency of metal removal using nitric acid, while the addition of hydrogen peroxide eliminated NO_x_ emission.

### 3.4. Leaching Efficiency

**Figure 7:**
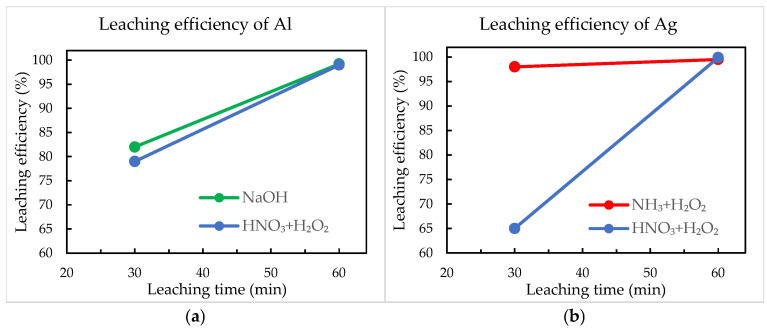
Leaching efficiencies of (**a**) aluminium and (**b**) silver.

The efficiency of aluminium and silver removal from crystalline silicon solar cells was quantified using SEM-EDS analysis by assessing the surface elemental composition before and after chemical treatment. The results are presented in Figure 7.

NaOH demonstrated effective selectivity for aluminium, achieving approximately 82% removal efficiency after 30 min, which improved significantly to nearly complete removal after extending the reaction time to 60 min. The rapid and selective aluminium dissolution confirms the effectiveness of alkaline leaching under mild conditions, supporting previous findings in solar cell recycling [17,29]. Selective silver leaching using an NH_3_ + H_2_O_2_ solution achieved notably high efficiencies, already within 30 min, reaching almost complete removal of Ag after 60 min. The simultaneous leaching method utilising a HNO_3_ + H_2_O_2_ solution initially showed comparatively lower performance, particularly for Al (79%) and Ag (65%) after 30 min. This initial lower efficiency could be due to slower dissolution kinetics or partial passivation effects on metal surfaces. However, extending the treatment duration to 60 min significantly improved results, achieving nearly complete aluminium and silver removal exceeding 99% of leaching efficiencies.

Overall, all three methods achieved high leaching efficiencies, but the sequential treatments (NaOH for Al and NH_3_ + H_2_O_2_ for Ag) provided faster initial metal dissolution. In contrast, simultaneous nitric acid-based leaching required extended treatment times to achieve nearly complete efficiencies, although it offered the possibility to extract both metals in a single step.

## 4. Discussion

This study investigated three hydrometallurgical leaching approaches for the recovery of aluminium and silver from crystalline silicon solar cells. While all methods achieved high overall leaching efficiency, they exhibited differences in terms of reagents employed, metal selectivity, and reaction behaviour. A comparison of the investigated methods with those discussed in the literature is presented in Table 1.

The sequential leaching route demonstrated rapid and selective metal removal under relatively mild reaction conditions. Aluminium was efficiently dissolved from the back-side contacts using a NaOH solution, achieving high efficiency (99%) after 60 min. Silver extraction with NH_3_ + H_2_O_2_ was similarly effective, reaching 98% efficiency at 30 min and 99% at 60 min. This two-step procedure offered precise control over each metal’s dissolution, reducing the potential for unwanted reactions between reagents and nontarget elements, and completely avoiding NO_x_ emissions, thus addressing environmental and occupational safety concerns associated with traditional nitric acid processes.

In comparison, the simultaneous method using HNO_3_ + H_2_O_2_ sought to simplify processing by extracting both metals concurrently. While initially, slower leaching kinetics were exhibited at 30 min, this approach ultimately provided superior overall metal recoveries at 60 min (99% Al and 99.9% Ag). These recovery efficiencies matched or even surpassed traditional nitric acid methods frequently reported in the literature [17,21,24]. The addition of H_2_O_2_ enhanced reaction efficiency and reduced reaction times compared to conventional processes. Furthermore, it proved effective in suppressing NO_x_ formation, as no visible gas emission was observed, demonstrating a significant environmental advantage over leaching with nitric acid alone.

When compared with recent alternative NO_x_-free silver leaching approaches (H_2_SO_4_ with sonication treatment or C_6_H_10_O_8_ + H_2_O_2_), the developed methods show distinct practical and operational advantages. Both sequential and simultaneous approaches utilise readily accessible and cost-effective inorganic reagents and are preferable in duration of the process. Conversely, other methods often rely on more complex procedures, costly processing, or additional purification steps. While alternative NO_x_-free strategies show promise, especially in terms of environmental impact, the cost and complexity may limit their scalability. The present study’s methods provide a balanced solution combining high efficiency, operational simplicity, and broad accessibility of reagents, which may better support the large-scale implementation.

Although this study demonstrated the high leaching efficiencies of all three methods, several limitations should be acknowledged. First, the experimental conditions were fixed, and further optimisation of variables such as reagent concentration, time, temperature, and solid/liquid ratio should be investigated for industrial scale implementation. Second, while SEM-EDS surface analysis effectively quantified leaching performance, more detailed chemical analyses of leachate solutions could provide complementary insight into metal dissolution dynamics. Third, the recovery of metals from leachate solutions was not addressed in this study, as the concentrations of aluminium and silver were low and not the focus of this work. However, future studies may explore concentration, precipitation and, exclusively for silver, chemical or electrochemical recovery strategies where appropriate.

## 5. Conclusions

This study evaluated three hydrometallurgical leaching methods for aluminium and silver recovery from crystalline silicon solar cells. The sequential method applying NaOH for aluminium followed by NH_3_ + H_2_O_2_ for silver provided selective and efficient metal extraction, reaching 99% for both metals within 60 min. The single step method employing HNO_3_ + H_2_O_2_ achieved similarly high efficiencies (99% aluminium, 99.9% silver), despite a slightly slower initial extraction efficiency. The sequential method stands out for its rapid and selective leaching under mild conditions, ideal for environmentally conscious metal recovery, while the simultaneous method offers a simpler alternative suitable for process streamlining. Both tested approaches successfully prevented NO_x_ emissions and offer a simple and cost-effective operational procedure. With the projected dominance of silicon-based photovoltaics in the global market, further development of such efficient, practical, and environmentally friendly recycling processes remains essential for the sustainable management of photovoltaic waste.

## Figures and Tables

**Table 1:** Comparative overview of leaching approaches for silver and aluminium from solar cells.

Method	Reagents	NO_x_ Emission	Leaching Efficiency	Complexity	Main Advantages	Main Limitations	Reference
Traditional HNO_3_	HNO_3_	Yes	91% (Al) 99% (Ag)	Simple	Well known, rapid, universal	Significant NO_x_ emission	[17,19,24,26]
H_2_SO_4_ + sonication	H_2_SO_4_	No	100% (Ag)	High	Common acid, room temperature	Need sonication, long (48 h)	[25]
C_6_H_10_O_8_ + H_2_O_2_	C_6_H_10_O_8_; H_2_O_2_	No	97.4% (Ag)	Moderate	Eco-friendly, less hazardous	Higher costs, slower kinetics	[32]
This study: sequential	NaOH; NH_3_; H_2_O_2_	No	99% (Al) 99% (Ag)	Moderate	Mild conditions, rapid	Two-step process, H_2_ formation	This study
This study: simultaneous	HNO_3_; H_2_O_2_	No	99% (Al) 99.9% (Ag)	Moderate	Single step, rapid, effective	Requires careful H_2_O_2_ addition	This study

## Data Availability

The original contributions presented in this study are included in the article. Further inquiries can be directed to the corresponding author.
